# Doctor-perceived-barriers to telephone clinics at KwaZulu-Natal hospitals during the COVID-19 pandemic

**DOI:** 10.4102/safp.v63i1.5334

**Published:** 2021-08-26

**Authors:** Lushen Pillay, Renata Govender, Somasundram Pillay

**Affiliations:** 1 Department of Gastroenterology, Princess Alexandra Hospital, Harlow, United Kingdom; 2 Department of Medicine, King Edward VIII Hospital, Durban, South Africa; 3 Nelson R Mandela School of Clinical Medicine, University of KwaZulu-Natal, Durban, South Africa

**Keywords:** telemedicine, telehealth, COVID-19, barriers to healthcare, social distancing

## Abstract

**Background:**

The coronavirus disease 2019 (COVID-19) pandemic has led to an unprecedented international emergency, resulting in a need to adapt the existing healthcare systems, in order to enable ongoing patient care despite the current disruptions. Telemedicine may be a viable option to continue hospital workflow, however there are barriers to its implementation. We set out to establish what barriers might exist and to assess the viability of teleclinics within the province KwaZulu-Natal (KZN), as perceived by doctors.

**Methods:**

This was a quantitative, observational, survey-based study targeted at medical doctors working in both the public as well as the private healthcare sector in University of KwaZulu-Natal (UKZN).

**Results:**

One hundred and forty-seven (147) responses were included. The majority (86%) of respondents felt that telemedicine could provide a useful means to continuing hospital workflow, however, only 47% believed that it was a viable option for their unit. The major barrier identified was a feeling that doctors would-be at-increased medico-legal risk. Only 38.4% of doctors were familiar with the Health Professions Council of South Africa (HPCSA) guidelines on telemedicine usage. Other major barriers included: doctors feeling uncomfortable with not seeing a patient in person or not being able to perform a thorough physical examination. Other reasons identified as potential barriers were doctors foreseeing difficulty in accessing patient medical records and the absence of available systems to order investigations without the patient being physically present.

**Conclusion:**

Telemedicine is currently not widely utilised in KZN; although most doctors were of the opinion that it could be a useful tool in order to continue the workflow during the pandemic. The major barrier identified were issues surrounding medico-legal coverage.

## Background

The coronavirus disease 2019 (COVID-19) pandemic has led to an unprecedented international emergency, resulting in a need to adapt the existing healthcare system to enable ongoing patient care despite the current disruptions. As a respiratory virus with droplet transmission, the core principle of infection control is social distancing to prevent transmission. In the initial phases, there were widespread ‘lockdowns’ that aimed to limit peoples’ movement, with South Africa having stringent restrictions, like many countries across the world. Hospitals worldwide have adapted by changing their policies and cancelling non-urgent medical appointments and services, resulting in a significant decline in patient numbers across all sectors apart from COVID-19 infections.^1^ Despite the easing of restrictions, social distancing continues, with most authorities recommending people to keep distance of 1 and 2 meters apart. The structure of outpatient healthcare will have to undoubtedly account for this change; and this brings with it many challenges, including massive backlogs in outpatient consultations.^2^

When we move into the post-COVID-19 era, there are bound to be changes in how clinics’ waiting rooms are structured to adhere to the social distancing guidelines. Telemedicine may provide an avenue to alleviate the actual number of patients needing to attend face-to-face consultations. As multiple countries across the world enter repeated ‘waves’ of infection, this remains relevant.^3^

Telemedicine is defined as ‘the remote diagnosis and treatment of patients utilizing telecommunications technology’.^4^ Its utilisation has increased through the COVID-19 pandemic to allow healthcare providers in many countries, to continue to treat their patients safely.^1^ Many authors have described virtual means of assessing patients with COVID-19 infection in order to limit face-to-face contact and possible viral transmission.^5^ In contrast, others have used telemedicine to continue outpatient management of patients with chronic illnesses. Multiple forms of telemedicine have been described; ranging from simple telephone clinics to video consultations and employing various new software technologies and applications that have been developed specifically for this purpose.^6^

While most systems require substantial start-up investment, telephone clinics are low-cost and an effective way of continuing to see outpatients. In its most basic form, the patient is contacted via telephone, and their clinical history is taken; if needed, investigations can be arranged, and prescriptions can be posted or emailed to the patient. The significant drawback is that no physical examination can occur, and patients need to adequately self-report the symptoms. Doctors also need to feel confident enough to make an appropriate assessment without having examined the patient or having had access to bedside investigations such as a glucometer reading or urine dipstick, in some circumstances. This is however not absolute, innovative means of having access to limited investigations maybe possible in some settings. It is also essential to have integrated means to arrange studies that may be lacking in lower resource settings – it has been the experience of the authors, that in many district level hospitals, x-rays are usually only done when the patient presents with a paper-based request form. We are likely to see differences amongst doctors of different levels of experience and what they believe to be barriers to telehealth; an experienced clinician is expected to be more comfortable consulting in a virtual assessment than an intern. To our knowledge, no South African data is available as to how different grades of doctors perceive telemedicine.

One of the significant advantages to telemedicine during the COVID-19 pandemic is that hospital workflow can continue, and patients with non-COVID-19 related illnesses can continue to receive medical treatment. International studies have shown that shifting face-to-face clinics to virtual clinics works well and allows outpatients to continue to receive care.^1^ A recent global study looked at telemedicine’s adoption in inflammatory bowel disease units during the pandemic. It showed that pre-pandemic over 75% of consultations were face-to-face. This has dropped to less than 25%, with over 50% of patients now being consulted over the telephone. The South African gastroenterologists who responded to this survey noted internet connectivity as the major barrier to telemedicine.^7^

In South Africa, the Health Professions Council of South Africa (HPCSA) is responsible for regulating medical affairs and practitioner conduct. At the start of the pandemic, the HPCSA published a guidance document which stated^8^:

Telehealth is only permissible in circumstances where there is an already established practitioner-patient relationship, except where Telepsychology and/or Telepsychiatry is involved, in which case telehealth is permissible even without an established practitioner-patient relationship. (p. 1)

This guideline had been widely panned by practitioner bodies for being overly restrictive and have stated that this guideline will limit telemedicine utilisation by practitioners. A revision was made in May 2020 stating^9^:

Telehealth should preferably be practiced in circumstances where there is an already established practitioner-patient relationship. Where such a relationship does not exist, practitioners may still consult using telehealth provided that such consultations are done in the best clinical interest of patients. (p. 1)

This policy change was supported by the Medical Protection Society (MPS), which has established that they will still provide liability cover for practitioners who engage in a virtual consultation.^10^ While doctors are legally entitled to run virtual clinics and have assurances from indemnity bodies, we sought to ascertain whether they felt confident enough with their liability coverage to run virtual clinics.

A systematic review published by Kruse et al. in 2018 identified numerous barriers across the world to the implementation of telemedicine services. In Africa, the three major obstacles were cost to the provider, language barriers, and patients not having access to a telephone. Other significant obstacles identified in multiple countries included: reimbursement of costs, legal liability, privacy and confidentiality concerns, and concerns around data security.^11^

While vast amounts of data are being published on COVID-19 and telemedicine across the world, to date there has been no published work looking at barriers to telemedicine in South Africa, with specific regard to COVID-19. The objectives of this study were to establish what the doctor-perceived barriers were in response to the implementation of telemedicine clinics in KwaZulu-Natal (KZN), the basis of which was based on a model of telephone follow-up of existing patients and then to:

- Evaluate differences in perceptions amongst doctors of different levels of experience.
- Evaluate the differences in perceptions between the public and private healthcare sectors.
- Evaluate if doctors were aware of current HPCSA guidelines regarding telemedicine and COVID-19.
- To determine, if doctors were currently utilising telemedicine in practice and in what form.
- To determine, if doctors believed that teleclinics are a viable way of maintaining hospital workflow with the current social distancing guidelines.
- To assess the viability of teleclinics across a range of specialties.

## Methods

This was a quantitative, observational survey-based study targeted at medical doctors working in both the public and private healthcare sector at hospital level in KZN. Doctors of all grades (interns, medical officers [MOs], registrars, and consultants) were included. We submitted the protocol to the University of KZN Biomedical Research Ethics Committee (BREC) for ethical approval. A survey comprising 27 questions was distributed online through ‘Google documents’ via the South African Medical Association (SAMA) group email platform, social media, and direct messaging to eligible doctors. Doctors who entered responses that met the exclusion criteria were automatically restricted from proceeding with the survey. Data of doctors working outside KZN and non-doctor healthcare workers were excluded. We endeavoured to distribute the survey to a wide array of doctors working in both urban and rural settings, and at different hospital levels. All responses were collected electronically before being stored securely, and data analysed. Data was compiled using Statistical Package for Social Sciences (SPSS) software and analysis of variance (ANOVA) was used for statistical differences. Each question regarding perceived barriers allowed an answer of ‘major barrier’, ‘somewhat of a barrier’ and not a ‘barrier’, which was subsequently assigned a numeric value of 1, 2, or 3 for statistical analysis.

## Results

A total of 147 responses were included, the demographics and work circumstances are described in Table 1.

**TABLE 1:** Demographic profile of respondents.

Variable	Group	*n*	%
Gender	Male	91	62
	Female	56	38
Doctor experience grade	Intern	45	31
	MO	57	39
	Registrar	21	14
	Consultant	24	16
Hospital setting	Rural	10	7
	Semi-urban	26	18
	Urban	111	75
Hospital level	District	36	25
	Secondary	38	26
	Tertiary	64	43
	Quaternary	9	6
Hospital sector	Public	134	91
	Private	13	9

MO, medical officers.

**FIGURE 1:**
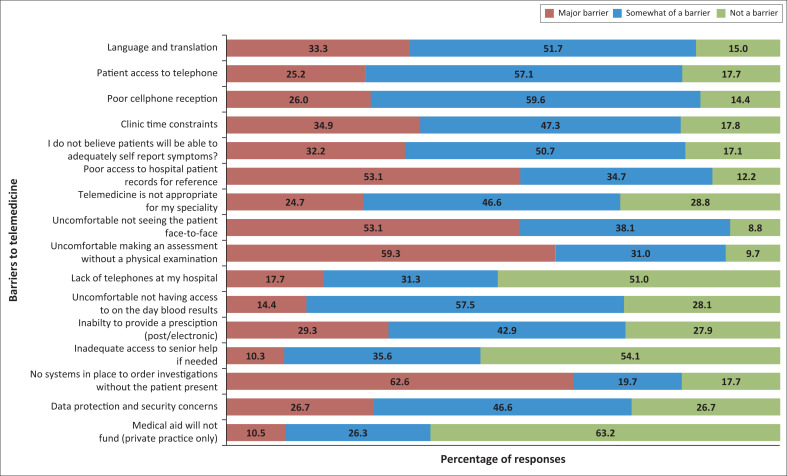
Doctor perceived barriers as per responses.

Figure 1 illustrates the response frequency to barrier questions posed to respondents, with answers recorded as being a barrier, somewhat of a barrier, or not a barrier.

**FIGURE 2:**
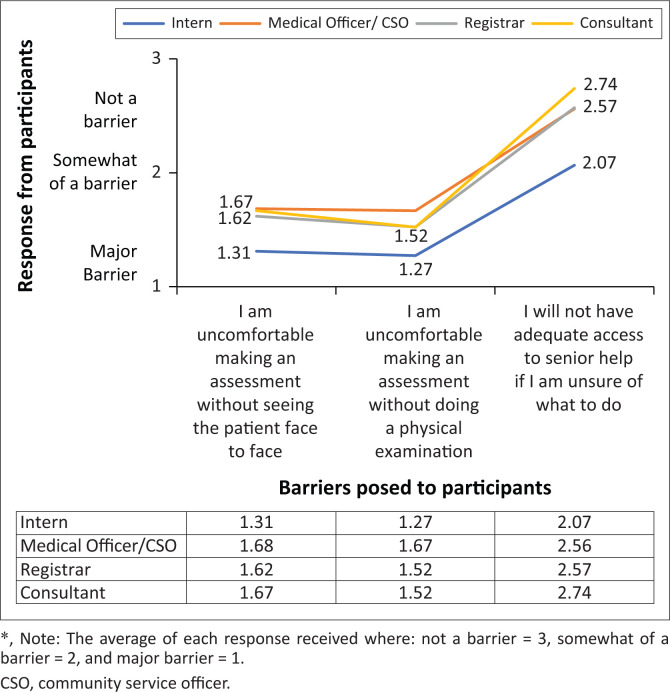
Responses showing significant difference between interns versus all other groups.

Figure 2 shows the three responses that met the statistical criteria for significance (ANOVA, *p* < 0.05) between each doctor’s grade. No significant difference was found between responses of MOs, registrars, and consultants. However, there was significance in three categories between interns and all other groups. This shows that the most junior doctors are relatively inexperienced and feel uncomfortable assessing a patient without seeing them face-to-face, these doctors also require senior supervision/input, which they feel will be less accessible when conducting a telephone consultation.

Table 2 demonstrates responses to questions of the HPCSA guidelines regarding telemedicine, current utilisation of telemedicine, and perceptions of viability of telemedicine.

**TABLE 2:** Responses to other questions.

Question	Yes (%)	No (%)
Are you familiar with HPCSA current guidelines on telemedicine during the pandemic?	38.4	61.6
If yes, do you believe these guidelines are too restrictive?	32.4	67.6
Do you feel confident that you will be covered medico-legally, if utilising telemedicine?	20.0	80.0
Does your unit already offer a telemedicine service?	19.7	80.3
Do you believe that telemedicine is a useful way to reduce hospital visits?	86.3	13.7
Do you feel that telemedicine is a viable option for the hospital that you work in?	47.3	52.7
Would you consider using telemedicine clinics for new patients attending a clinic?	24.0	76.0

HPCSA, Health Professions Council of South Africa.

**FIGURE 3:**
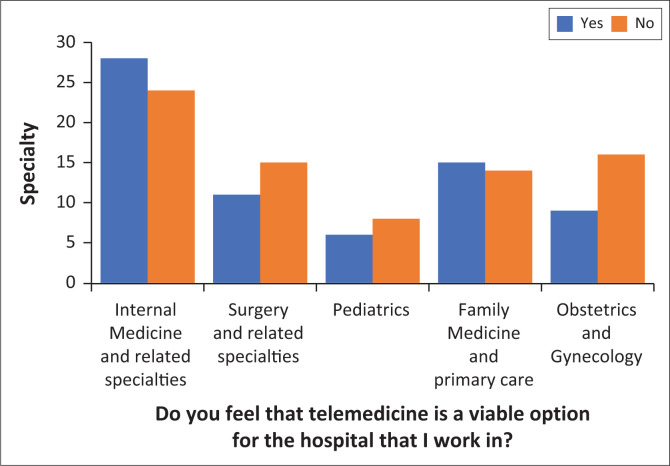
Perceptions of viability of telemedicine across specialties

Figure 3 shows the perception of the viability of telemedicine amongst doctors working in different specialties.

Table 3 illustrates the proportion of respondents who utilised paper-based medical records versus electronic medical records, and the perceived viability of telemedicine amongst each group.

**TABLE 3:** Perception of viability based on type of medical records used.

Question to practitioners	Type of medical records	Responses	Yes		No	Difference
			*n*	%		
What form of electronic records does your hospital use?	Paper-based	129	-	-	-	-
	Electronic	16	-	-	-	-
Do you believe telemedicine is a viable option for the hospital you work at?						
Type of medical records used:	Paper-based	-	56	43	73	*p* = 0.004
	Electronic	-	13	81	3	-

## Discussion

We gathered responses from a wide range of medical practitioners from across the province of KZN, and this ensured representation from various grades and settings as described in Figure 1. The majority of responses for this study originated from interns and MOs, this reflects the proportion of doctors of different grades working in hospitals, that is, a greater number of more junior doctors. Most responses stemmed from doctors working within urban settings and reflected the larger hospitals in these settings.

We looked at the current utilisation of telemedicine amongst doctors in KZN, and found that over 80% of the doctors who responded to our survey did not utilise telemedicine within the services in which they work. This showed us that the rate of utilisation of telemedicine is extremely low amongst the doctor who responded to our survey. Despite the current low uptake of telemedicine, over 86% of respondents said that they believed that teleclinics would have been a useful way to reduce hospital visits during the COVID-19 pandemic, clearly indicating that doctors feel that telemedicine would provide a meaningful way of continuing workflow. This is not surprising given the positive results that other countries have had in utilising telemedicine. However, despite over 86% of respondents believing telemedicine to be beneficial, only 47% believed that telemedicine was a viable option within the setting in which they work. This large discrepancy between belief in theory and implementation shows that there are significant barriers to utilising telemedicine in the province. The rest of this discussion focuses on what these barriers are, and by elucidation, is the first step in overcoming these barriers and perhaps moving closer towards possible implementation in the future.

Figure 1 illustrated the responses to the question as to what doctors found to be barriers to telemedicine. We found that language and the need for translation is a major barrier to telemedicine and in a country with 11 official languages, while translation problems would occur with face-to-face visits too, it is likely to be easier to provide translation services in person rather than over the phone. Patients either not having access to telephones or having poor cellular reception were also considered to be major barriers. This highlights the fact that many South Africans still live below the poverty line or are found in remote areas. Time constraints was another major barrier described and serves to reflect the inherent problem of limited numbers of doctors working within the public healthcare system.

Doctors also reported that they felt that their patients would not be able to adequately self-report symptoms. For example, a patient may not be able to fully describe a rash or a mass, which the doctor would then not be able to see for themselves, as is the case with face-to-face clinics. This finding ties in with language barriers and with some patients having lower levels of education. This is a difficult barrier to overcome as it is multifactorial and a problem in most low-to-middle income countries.^12^

Poor access to hospital records pose a major barrier as all, but a few public hospitals, still use paper-based records. This system usually relies on the patient collecting their hospital record prior to having a consult. More administrative staff is needed to overcome this, something which is not always possible in a resource-limited setting. Hospitals utilising electronic patient records systems are able to adapt to and perform telemedicine more easily. We found that doctors already utilising electronic patient records felt that telemedicine was more viable than those using paper-based systems (Table 3). We noted that most doctors felt that the hospitals at which they worked at had adequate telephones to facilitate telephone clinics.

Not having access to on-the-day blood results was noted to be another barrier. This reflects the reliance that healthcare professionals place on laboratory investigations, and is a notable problem encountered in junior doctors who rely on blood results in cases of diagnostic doubt. Almost three-quarters (72.2%) of respondents noted that the inability to provide a prescription was a barrier. In the public sector, patients usually collect medications from the hospital they attend and need to present their hospital chart. Methods of being able to post out prescriptions or systems utilising electronic prescription would be needed to overcome this. Hospitals not having systems to be able to order investigations without the patient being physically there, was perceived as a significant barrier as reported by 82.3% of our respondents. Data protection and digital security concerns were not considered as major barriers to telemedicine according to our respondents.

The HPCSA regulates the practice of medicine within South Africa and as such, it is essential that practitioners are aware of what the ethical and legal confines of telemedicine are. We found that in our cohort of doctors, only 38.4% (Table 2) of respondents were aware of the latest HPCSA guidelines regarding telemedicine. This is possibly due to doctors not currently using telemedicine and thus these guidelines were not relevant to them. It could also imply that more widespread dissemination of guidelines to ‘on the ground’ staff is needed. Overall, most of those doctors who were aware of what the guidelines entailed did not find them to be overly restrictive. One of the key issues raised in the guidelines is that there is a preference towards a pre-existing doctor-patient relationship with users of telemedicine, this stream of thought was supported by majority of our cohort, as only 24% of doctors would consider using telemedicine for first time clinic attenders. This is likely to be related to the complexities of making an initial assessment when a more in-depth examination and multiple investigations might be needed in order to make a diagnosis versus a follow-up clinic review in which a response to treatment is being assessed.

As medicine becomes more defensive, medico-legal implications of practice are of increasing concern to doctors. This is no different in terms of telemedicine, with 80% of our respondents indicating that they felt that they would not be covered from a medico-legal standpoint This was the most significant barrier identified in our study. This, even though the MPS does in fact make provision for telemedical consultations. As more practitioners use telemedicine, it remains to be seen what litigation arises as a result.

When looking at doctor perceptions, it was essential to look at how the perceptions of different grades of doctors differ. We found that of the questions posed to doctors, only three barriers showed significant difference between the different grades of practitioners (Figure 2). This difference was significant when it was applied between interns compared to MOs, registrars, and consultants. Considerably more interns felt uncomfortable with making an assessment without face-to-face consultation or without conducting a physical exam when compared to all other grades of doctors. These results are not surprising as junior doctors are unlikely to have acquired sufficient skills as interns to allow them to make an adequate assessment without a physical examination. Certainly, when it comes down to more complex issues in sub-specialty medicine, an intern would not have the same level of experience as a full-time medical officer or registrar. Another major barrier identified by interns was that they would not have adequate help if needed, as it is less practical to pass a telephone over from the junior to senior doctor than it would be for a senior to walk into an examination clinic room to assess a patient. It is clear from our study that when planning to implement teleclinics, it should be aimed at the more senior members of the medical team. If interns are to be utilised to conduct these clinics, they will require more intensive training, supervision, and support. With current intern rotations lasting for a maximum of 4 months, this would lead to a significant gap in service delivery as training will need to occur with each rotation and this might not be ideal in sustaining workflow. In the longer term perhaps, what is needed here is the introduction of undergraduate modules dealing with telehealth and telemedicine and methods detailing how to introduce this into routine clinical practice. This may give newly qualified doctors a greater sense of empowerment to take on this role.

We compared the perceptions of viability of telemedicine amongst doctors of different specialities and found no significant difference between those working in predominantly medical (internal medicine, family medicine, and paediatrics) versus those in surgical specialities, obstetrics, and gynaecology.

### Limitations of study

Unfortunately, there was a paucity of responses from private practitioners and therefore this resulted in us not being able to make meaningful distinctions between both healthcare sectors. We only looked at the perceptions of doctors, and in future it would be necessary to include other stakeholders when further evaluating the viability of telemedicine.

## Conclusion

We have found that telemedicine currently is not widely utilised in KZN, although most doctors do believe it could be a useful option to continue workflow during the pandemic. Despite this however, most felt it would not be viable for the hospital they worked at. The major barrier to implementation identified are issues surrounding medico-legal coverage for doctors. Other barriers relate to the utilisation of paper-based medical records and investigation ordering systems, which rely on patients being present in the hospital. Junior doctors were shown to be more apprehensive about using telemedicine and would require intensive training, support, and supervision. The government needs to heed results of such studies to develop strategies to implement relevant intervention plans.
